# The impact of services that offer individualised funds, shared management, person-centred relationships, and self-direction on the lived experiences of consumers with mental illness

**DOI:** 10.1186/1752-4458-8-20

**Published:** 2014-06-03

**Authors:** Sunila Peterson, Angus Buchanan, Torbjorn Falkmer

**Affiliations:** 1 School of Occupational Therapy and Social Work, Faculty of Health Sciences, Curtin University, Perth, Western Australia, Australia; 2 Department of Medicine and Health Sciences (IMH), Rehabilitation Medicine, Faculty of Health Sciences, Linköping University & Pain and Rehabilitation Centre, UHL, County Council, Linköping, Sweden; 3 School of Occupational Therapy, La Trobe University, Melbourne, Vic, Australia

**Keywords:** Consistent and quality support relationships, Corrective emotional experiences, Grounded theory

## Abstract

**Background:**

Mental health service providers across Australia, including Western Australia (WA), have begun to offer individualised funds, shared management, person-centred and self-directed (SPS) services. No research exists on the impact of SPS services on the lived experiences of these particular consumers. This study explored the impact of a SPS service offered for the first time in WA to consumers with mental illness.

**Methods:**

Data on sixteen consumers’ lived experiences were analysed using an abbreviated grounded theory approach. These data had been developed by the consumers, Guides (staff) and an independent evaluator, and most of it had been collected in the past prior to the commencement of the study.

**Results:**

Three over-arching categories, and related subcategories, emerged indicating that 1) access to individualised funds enabled practical and psychological benefits to consumers; 2) consistent contact in shared management and person-centred relationships enhanced the provision of timely and meaningful staff support to consumers; and 3) high quality shared management and person-centred relationships with staff and the opportunity to self-direct enabled consumers’ change and growth.

**Conclusions:**

SPS services enhanced consumers’ lived experiences and enabled staff to provide and consumers to experience timely access to recovery resources, consistent contact, responsive and high quality support, and self-direction of services. In this, consumers changed, grew and achieved desired recovery experiences. The overall impact of the SPS service seemed to be founded on the goodness of fit between person characteristics of staff and consumers, which enabled rich support that provided for corrective emotional experiences. This enabled consumers to build meaningful and hopeful lives where they started to live with, and beyond, their mental illness.

## Background

People with mental illness are at risk for experiencing serious socioeconomic [1-3] and health service challenges [4]. Over the past century, a biomedical model service approach has dominated health care services [5,6]. This approach views mental illness as a disease of biological origin, and effective treatment as being curative of the disease [7,8]. The biomedical approach has promoted, for some, the acceptance of mental illness as being the cause of biological, rather than an inherent personal flaw [7]; however this acceptance may be restricted to a specific disorder and/or the engagement of treatment, rather than acceptance of the person living with mental illness [9]. Further, this approach has provided an invaluable and empirical sound methodology for understanding mental illness; yet it is criticised for not adequately meeting consumers of mental health services’ recovery needs [6,7].

The biomedical model approach is commonly criticised as being reductionistic for viewing mental health as the complete absence of illness, and consumers as being ‘passive patients and victims of circumstances’ who have little or no responsibility in the presence and/or cause of the illness [6,8,10-12]. Consumers’ needs are often understood in relation to aspects of the mental illness (e.g., diagnosis, deficits, relapse, risk-management and treatment matters). They are required to cooperate and comply rather than actively participate in determining their treatment with health care professionals. Consumers report that a biomedical model approach is too rigid, top-down driven and focused on meeting professionals’ but not their own needs, which isolates and disempowers them [10,13,14]. In contrast, consumers’ needs commonly relate to aspects of life such as work, education, spirituality, pets, interpersonal relationships and health. Furthermore, they desire equality, responsibility, flexibility and control in their support relationships and recovery [10,13,15]. Thus, a biomedical model based service has limited capacity to adequately meet such consumer needs.

Recovery may be viewed as a personal journey that consumers make over time, where they move towards building a fulfilling and meaningful life for themselves, living with and beyond their mental illness to experience a positive sense of identity founded on hope and self-determination [12,16,17]. Within this context, biomedical model based services are unable to adequately meet consumers’ recovery needs [5,10,16]. Thus, with mental illness continuing, consumers’ dissatisfaction from not having their needs met, beside improved practices in the disability sector, services are moving from biomedical model based approaches to more consumer focused ones [12,17,18].

One type of consumer focused services is based on a self-directed model of service [13,15]. It provides shared management, person-centred and self-directed (SPS) services to consumers, with an individualised funding component. Here, consumers and service staff (e.g., broker, advisor, guide, mentor, or support worker) work together in shared management relationships, where each party is responsible for the consumers’ undertaking of the service, including the spending of funds [19]. Individualised funds may also be known as personal budgets and direct payments [13,15,20,21]. These funds are given to consumers to spend on resources (goods and/or services) that they view as important for their recovery, which are approved for procurement by the service provider [13,15,20,21]. These funds may be directly paid to and administered by consumers or a delegate chosen by consumers (e.g., the service provider), where consumers and staff work collaboratively to reconcile expenditure [13,15,20,21]. In shared management relationships staff support consumers to use their expertise and strengthen their capacity to self-direct their recovery journey [1,10,15]. Shared management relationships complement the provision of person-centred relationships. Both types of relationships place consumers at the centre and in control of managing the SPS service and their recovery [10,13,15,22]. In these relationships, staff encourage consumers to identify and define their recovery needs, goals and resources. They support consumers to make decisions and take actions, using their individualised funds, to procure resources and progress their recovery [13,15]. In doing this, consumers work with known others (e.g., service staff, family and friends) and form new and/or improve existing connections with individuals, groups or organisations for recreational, social and/or professional purposes [3,12,13].

Thus, SPS services with individualised funds enable consumers to identify recovery needs and resources across all aspects of their life, and not just in relation to their mental illness as is often the case with services based on a biomedical model. SPS services enable them to have funds to procure new, or continue to use, current resources to meet health and fitness, utilities, home and equipment, and psychological, social and/or recreational needs [1,13,15].

In SPS services, the quality of relationships formed between consumers and staff impact on their service experiences. Greater time invested in consumer-staff relationships enables consumers to develop and implement their recovery plans and self-direct services [13,15]. Moreover, the quality of these relationships impact on consumers’ satisfaction, trust and recovery experiences [10,13,15]. Staffs engagement in active listening, holistic views of consumers’ needs, and respect for consumers’ freedom, choice, responsibility and reciprocity enhances the quality of relationships [10]. The quality of relationships is also influenced by past interpersonal relationship experiences of both consumers and staff, and consumers’ needs and abilities and willingness to actively participate [23-27]. Also, staffs’ capacity to be empathic, sensitive, and aware of process matters, consumer experiences, and their own reactions and experiences impact on the quality of relationships [25]. Further, staffs’ ability to communicate openly, honestly and directly also moderates the quality of relationships [25].

Such staff characteristics support consumers to progress their recovery and self-direction, enabling them to work through maladaptive relational patterns and challenges [23,25,26]. These characteristics also enable for the establishment of clear relationship boundaries, support of consumers’ autonomy and independence [27], and an effective shared management relationship process. Furthermore, these characteristics also impact on the usefulness of relationships, influencing the development of safety, trust, and the opportunity for consumers to encounter corrective emotional experiences [23,26]. Corrective emotional experiences provide consumers with the opportunity to encounter relationships in new and enabling ways, which offers them a chance to re-learn and/or expand their experience of past relational patterns [26]. Consistent exposure to corrective emotional experiences support consumers to develop more helpful internal working models of themselves and their relationships with others and their world, which may enhance their motivation, growth and recovery experiences [25,26,28].

SPS services that provide consumers access to individualised funds and consistent and high quality shared management and person-centred relationships offer consumers the opportunity to self-direct their recovery journey to meet their life needs [1,13-15,20,21,27,28]. Recently, service providers across the Australian mental health sector, as have service providers in the UK and USA for the last 15 to 20 years, are introducing SPS services to consumers living with mental illness through policy and practice change [3,22,29].

In the state of Western Australia (WA) a non-denominational community benefit service provider of mental health services was one of the first to offer SPS services to consumers with mental illness. They invited sixteen consumers to participate in a pilot SPS service over a selection period of two to three weeks. All consumers accepted the invitation, and undertook the SPS service in three groups (of five to six consumers) where each group commenced services approximately four to six months apart over a total period of sixteen months.

Consumers first attended an information session followed by five fortnightly small group person-centred planning (PCP) sessions over three months. Here, consumers worked with service providers’ staff (e.g., PCP facilitators, support workers, and two Guides), friends and/or family to develop their ‘future dreams’. For the next twelve months they moved towards achieving these dreams working in one-to-one relationships with one of two Guides. The Guides were responsible for providing consistent shared management and person-centred relationships to consumers, from selection, through the PCP process, and across the entirety of the service. The Guides provided the most concentrated levels of support after the PCP process. Here, they visited consumers weekly, for two to six weeks to support them to develop their dreams into recovery goals on their action plans. These action plans were then reviewed and endorsed for individualised funding by the service provider; yet consumers had the flexibility to change their action plans at a later time as needed to accommodate unpredictable circumstances. Once consumers received their individualised funding allocation, the Guides visited them fortnightly to support them to implement their action plans. The action plans provided consumers with a map from which they navigated their recovery journey throughout the service. Gradually over time the Guides reduced the provision of support and engaged support workers to provide specific support to consumers as they needed. The Guides then took on more of the guiding role they had consistently provided, to continue to support consumers to understand what was expected of them to successfully implement their action plans, reconcile expenditure of their individualised funds, and/or prepare for PCP reunions. As during the PCP process, the consumers also had access to support from other service provider staff (e.g., support workers or SPS service managers), family and friends, and individuals and/or groups in the community throughout the service.

Research from the UK and USA suggest that SPS services seem to provide meaningful support that effectively meets consumers’ needs. However, little is known about the impact of these services, which have only recently being introduced across Australia, on the lived experiences of consumers with mental illness living in WA. The service provider who had offered SPS services for the first time use of consumers with mental illness in WA engaged the authors to evaluate and develop a pilot model of SPS service practice for use across the local mental health sector. The present study focused on one section of this pilot model that related to the aim of this study. It explored the impact of the SPS service on the lived experience of consumers with mental illness using data developed by the consumers, alongside others, to gain a deeper and holistic understanding [30,31].

### Aim

This study explored the impact of SPS service components, including access to individualised funds, shared management, person-centred relationships, and the opportunity to self-direct their services on the lived experiences of consumers.

## Methods

### Design

This study analysed data that had been compiled in the past, which at that time captured both retrospective and current experiences (Additional file 1) of sixteen consumers with mental illness while they were undertaking a SPS service. This method is also reported elsewhere [28].

## Materials

The sixteen consumers, who had undertaken the SPS service, self-selected into the study and consented for researchers to have access to data, most of which had been developed prior to the study. The data included information relating to individualised funding and hours of support provided to the consumers, with the latter being the only data collected (retrospectively) at the time of the study. In addition, 473 documents of data (Additional file 1) relating to consumers’ lived experiences were also collected. Lived experiences of consumers was defined as the accounts of their life experiences living with, and beyond, mental illness prior to and while they were undertaking the SPS service [12]. The data had been developed in the past by the sixteen consumers, two Guides and another evaluator. The other evaluator, who was independent of the researchers/authors, had been engaged three times by the service provider (prior to the study) to evaluate the PCP process of the SPS service. This independent evaluator had interviewed consumers, and two Guides and support workers, and used their feedback to develop evaluation reports on the PCP process.

Information on individualised funding and hours of support was provided to the researchers by the Guides and the SPS service manager. Further, the 473 documents of data, collected in the past and held by the service provider and the independent evaluator, were de-identified and provided to the researchers. The Guides provided consumers’ action plans, two questionnaires (‘Most Important Changes (MIC) to My Life’ completed one to four times and ‘Recovery’) that had been completed for the independent evaluator, and PCP reunion speeches. They also provided documents on their own personal learning, reflections on consumers’ progress and services aspects (completed up to four to seven times), meeting minutes, and own reunion speeches. The independent evaluator provided the consumers’ and staff interview data reports and three evaluation reports and related summaries. The data developed by these three sources varied in the type (e.g., background, experiences around living with mental illness, hopes, and experience of the SPS service) and style of documentation (e.g., bullet point, short answer, Likert scale, or narrative) as described in Additional file 1, and this influenced the reporting of some of the results.

### Recruitment of consumers and their characteristics

Twelve of the sixteen consumers were using other services from the service provider prior to starting the SPS service, while four consumers were new referrals to the service provider. The Guides and/or other staff (e.g., SPS service managers or support workers) invited potential consumers that they thought might be ready to undertake the SPS service. They visited potential consumers at their homes, discussed aspects of the SPS service, and responded to their questions. All sixteen consumers who were invited to use the SPS service contacted the Guides to progress their recruitment and also consented, at a later time, to participate in the present study; i.e., they self-selected into the study. At the time of the study, although seven consumers had undertaken and completed the SPS service, they were keeping in regular contact with the Guides. Three consumers were still implementing their action plans some sixteen months since commencing the SPS service. The last group consumers (*n =* 6) who had commenced the SPS service six months previously had recently begun to implement their action plans.

The data showed that the overall mean age of consumers was 46 years. The majority of consumers were female (56%) and Caucasian (94%), with one consumer being an Australian Aboriginal person (Table 1).

**Table 1:** Consumers’ age, ethnicity, educational and mental illness experiences

**Demographics**		**Measurement**
Age		Mean years
	Overall (*n =* 16)	46 years
	Female (*n =* 9)	48 years
	Male (*n =* 7)	45 years
Ethnicity		Number and Percentage
	Caucasian	*n =* 15 (94%)
	Australian Aboriginal	*n* = 1 (6%)
Experience of educational attainment		
	Did not complete secondary school	*n* = 7 (44%)
	Completed secondary school	*n* = 4 (25%)
	Unknown	*n* = 5 (31%)
Experience of mental illness		
	Depression*	*n* = 7 (44%)
	Schizophrenia	*n* = 7 (44%)
	Bi-polar disorder*	*n* = 2 (13%)
	Generalized anxiety disorder	*n* = 2 (13%)
	Post-traumatic stress disorder	*n* = 1 (6%)
	Delusional disorder	*n* = 1 (6%)
	Substance-induced psychotic disorder	*n* = 1 (6%)

*Specific disorder as per the Diagnostic and Statistical Manual –Fourth ED–Text Revised is unknown.

The demographic details on consumers were gleaned from the data that were available, limiting meaningful comparisons with relevant national statistics. It was noted that the majority of consumers (87%) were in older age groups (40 to 64 years) compared with national statistics that showed younger age groups (16 to 35 years) as having higher rates of mental disorders [32]. Many consumers had not completed secondary school although five, including two who had not completed secondary school, had completed higher education and/or training. Five consumers were living with two concurrent mental illnesses, and the mental illnesses diagnosed across consumers related to depressive and/or substance induced psychotic disorders, and single incidences of anxiety, stress and delusional disorders. The consumers’ diagnosis of mental illness is consistent with the broader categories of disorders in national statistics (i.e., affective, anxiety and/or substance abuse); but meaningful comparisons (e.g., prevalence by disorder and/or gender) could not be determined due to data limitations [32].

The data showed that thirteen consumers had in the past occupied a range of jobs and roles across a number of industries, with some having held multiple occupations (e.g., employment, community services and parenting) at one time (Table 2).

**Table 2:** Consumers’ historical and contemporary occupational experiences

**Occupational experiences**		**Number and percentage**
**Past occupations worked in**		
	Parenting (i.e., children and/or grandchildren)	*n =* 10 (63%)
	Customer services (i.e., retail, diesel service, cleaner, store man, receptionist, inspector at an animal welfare agency, general office/domestic work, sheltered workshop, and shop work)	*n =* 7 (44%)
	Transport and logistics (i.e., truck driving, fleet management and accounting)	*n =* 3 (19%)
	Community (i.e., Aboriginal community work, youth outreach, fundraising, and mental health services)	*n =* 3 (19%)
	Government (i.e., hospitals and education department or school)	*n =* 2 (13%)
	Unknown	*n =* 3 (19%)
**Past occupational roles occupied**		
	Parent	*n =* 10 (63%)
	Frontline (i.e., customer service and/or administrative)	*n =* 6 (38%)
	Management other business (i.e., manager, assistant manager, or supervisor)	*n =* 4 (25%)
	Management own business (i.e., transport, retail, art, or writing)	*n =* 3 (19%)
	Advocating, facilitating or coordinating (i.e., volunteer or social justice work)	*n =* 2 (13%)
**Current work occupational status**		
	Unemployed	*n =* 8 (50%)
	Employed	*n =* 2 (13%)
	Unknown	*n =* 6 (37%)

At the time of the SPS service, eight consumers were unemployed due to physical (e.g., back or respiratory) and/or mental health problems, not having adequate skills, and/or not being able to find suitable work. Two were employed. The employment status of six consumers was unknown. All consumers were struggling financially and renting, with some sharing accommodation. Nine consumers were renting privately, seven were renting subsidised housing, ten were sharing accommodation with their children, partner and/or family and six were living alone. Within limits of the data these consumers’ socio-economic experiences seemed comparable with national statistics that suggests people living with a higher prevalence of mental illness experience socio-economic disadvantage; yet meaningful comparisons were restricted [32].

### Analysis

This study analysed the individualised funding expenditure data and hours of support using Excel spreadsheets. The 473 documents (760 pages) of data relating to lived experiences of consumers were coded using NVivo 9.2 (QSR International®, Victoria, Australia) qualitative software. Coding was conducted using the key tenants of an abbreviated grounded theory approach [33,34]. This enabled the evaluation project aim of developing a pilot SPS framework to be achieved. All types of data from all sources were coded to ensure that the meanings embedded in these were not missed. However, this led to higher percentage of documentation being coded until saturation of categories was achieved (89.05%).

All of the consumers’ data were coded; although saturation of codes was achieved at 79.81%. Consumers’ data were coded first to prevent the coding being influenced by the data from other sources. The Guides’ data were coded next followed by the independent evaluator’s data until the saturation of codes was achieved (Additional file 2). The volume of consumers’ data (285 pages of full writing and 21 pages of three quarters of a page of writing) was greater than the independent evaluator’s data (78 pages of full writing), which was greater than that of the Guides’ data (24 pages of full writing, and 339 pages of one quarter page of writing). More detail relating to the volume and saturation of data may be seen in Additional file 2. To support a grounded theory analysis approach [33,34] the literature review for the study was not conducted until after the data analysis. A bottom-up, all inclusive, systematic, open and focused coding approach was used to capture the meaning of experiences embedded within the data across physical, physiological, psychological, social, environmental and/or spiritual levels. Thus, the data drove the development of codes and allowed statements to be shared across codes if they reflected the relevant experiences. During coding, a constant comparative method was used. This ensured that the coder remained mindful of the potential meanings of the codes, and similarities, differences, and relationships between the codes, which were documented in memos and annotations.

Over eight weeks of coding, 10% of codes established at any point in time were randomly selected (four times), using the Excel spreadsheet ‘*randombetween’* function, for quality checking by the chief investigator and an independent research officer of the evaluation project. The meaningfulness of statements within these codes was assessed, and the quality checkers and coder discussed and resolved any discrepancies by un-coding and/or re-coding statements to achieve consensus. Quality checking of a total of 26 codes and 240 statements showed an overall 96.25% agreement between the coder and quality checkers. Over the next two weeks coding was completed and the codes that had emerged were reviewed and compared. Codes that were duplicated were merged, resulting in a final set of 242 initial codes, which were quality checked achieving 99.6% agreement. Any discrepancies were discussed and resolved; where the final 242 codes that were retained had achieved 100% consensus.

The codes were then reviewed and compared and sorted several times, using a bottom-up approach. In this, highly related codes were grouped together to develop categories. Categories were then grouped together to develop higher categories. The categories were reviewed and compared within NVivo 9.2. Memos, annotations and maps developed using Microsoft VISIO software helped to define similarities, differences and relationships between categories (within and across tiers). This led to the development of a preliminary pilot model of SPS service practice. This model was reviewed by the chief investigator, and a reference group involving a consumer living with mental illness, the independent evaluator, a consultant who administered the PCP process, and three managers who worked in three different mental health services. No changes were required. Thus, the preliminary pilot model of SPS service practice was finalised and retained. Within this model, the section that related to the aim of this study, showed a three tiered categorical structure. This included three over-arching categories (first tier) founded on a varied number of sub-categories (second or third tiers) that had been established from the final set of 242 initial codes.

After the completion of grounded theory analysis and establishment of the model, the statements within categories were closely reviewed to ensure that their meanings within the context of their category were understood. In doing this, all key meanings of statements were documented as ‘data descriptors’. For example a statement ‘*using some funding to go on holiday planned for later this year’* sitting within the category *future aspirations* would be assigned data descriptors of *money, planning, and travel*. The use of data descriptors enabled all key meanings of statements embedded in large volumes of data within categories to be known. Without documenting data descriptors there was a risk that only the most obvious meanings of statements within categories would be noticed. Using the previous example, without data descriptors the following meaning may be derived: *consumers showed future aspirations and this related to travel*. The use of data descriptors allowed statements within categories to be systematically reviewed and all key meanings (data descriptors) to be communicated in full within the context of the categories they resided in. Using the previous example, the use of data descriptors may enable the following meaning to be derived: *future aspirations was supported from having access to funds, the ability to plan, and related to travel.* The data descriptors for categories were documented onto Excel spreadsheets and this information was used, alongside categories and related quotes, to present the results.

### Ethics approval

Ethical approval (OTSW-15-2011) was gained from the Office of Research and Development Human Research Ethics Committee at Curtin University in WA.

## Results

The three over-arching categories that were established suggested that the SPS service impacted on consumers’ self-direction of services and experiences. The first over-arching category related to the impact of the individualised funds on consumers. The two remaining over-arching categories related to the impact of shared-management and person-centred relationships and opportunity to self-direct services on consumers. These results were reported at a group level, rather than case level, using the terms that are defined in Table 3.

**Table 3:** Defining the amount of consumers reported on throughout the results section

**Amount of consumers**	**Number of consumers**
All	16
Most	15 – 11
Many	10 – 6
Some/few	5 – 1

### Over-arching category 1 - Access to individualised funds enabled practical and psychological benefits to consumers

Review of 183 statements from the consumers’ action plans and speeches data, independent evaluators’ interview data, Guides’ reflection data, and financial data indicated that access to individualised funds provided practical and psychological benefits to consumers. Over-arching category 1 was established on two subcategories. These included the: 1) costs and types of recovery activities procured; and 2) the impact of having access to individualised funds.

Consumers’ action plans and financial data showed that a wide range of recovery resources (goods and/or services) were planned for procurement with the use of the individualised funds (Table 4). These activities spanned across consumers’ whole of life needs and were valued by them as being important for their recovery.

**Table 4:** Types of recovery resources that consumers identified for procurement on their action plans and/or procured

**Activities (*n =*** **117) engaged to achieve recovery goals**	**Percent procured**
Developing skills and/or knowledge (e.g., getting a license or education and attending courses to learn to use computers, photography, massage, and painting)	26%
Purchasing equipment (e.g., computer, camera, gardening, kitchenware, TV antenna, and paint)	26%
Joining a group for social, health and fitness, and recreational purposes (e.g., dating sites, social groups, gymnasiums and health and fitness centres, model building clubs, and photography clubs)	21%
Developing aspects of ‘the self’ by doing courses (e.g., communication, confidence and assertiveness skills), securing counselling services for themselves and/or family members (e.g., individual or family counselling), and/or joining groups that could assist with discovering their heritage	16%
Taking time out (e.g., holiday, trip for fun and/or socialising, and enhancing connections with family who live overseas) with and/or without family	7%
Attaining certifications (e.g., first aid, working with children check and National Police Clearance)	3%
Other (e.g., securing a sitter to watch over dependents)	1%

The independent evaluator’s staff interview and nine consumers’ (denoted by C) speeches data showed that access to individualised funds was highly valued as it enabled consumers to procure resources important for their recovery that for many may not have been achievable without the funds. These consumers reported gaining practical benefits from having access to individualised funds, as highlighted in these statements:

“…started [study] in Aged Care…may achieve…academic ambitions sooner!” (C3)

“..thanks to individualised funding…doing an online course on computing…recapture and improve… skills…given me…opportunity…” (C4)

“…the funding…helped…[purchase] a bike…ride three times a week…” (C9)

These consumers also revealed that their motivation and feelings of self-worth and hope (for themselves or their children) were facilitated from having access to individualised funds, as stated:

“…being trusted with the money meant a lot…” (C1)

“…individual funding…confidence, self-discipline and self-esteem of…children…me… helped…” (C5)

“…the money to…return to the workforce…changed my outlook, wellbeing…helped me to take another look at life…join and participate in life…instead of hiding from it…” (C6)

The independent evaluator’s interviews and the Guides’ reflections data revealed that some consumers experienced initial challenges in managing their individualised funds. A few consumers did not keep receipts to effectively reconcile expenditure, purchased resources (e.g., incidentals) not identified on their action plans, and/or did not seek to gain value for money (e.g., choosing the most expensive). The Guides worked with these consumers to develop effective budgeting practices. Consumers were engaged to understand the value of keeping receipts and/or learn how to gain value for money when procuring recovery goods and/or services. They were also engaged to pay back money for expenditure that had occurred beyond action plan goals and/or learn how to manage limited money to procure expensive resources (e.g., using half of the individualised funds and half from other personal income). Some consumers from groups two and three had experienced disappointment at the beginning of service when they did not receive funding allocated amounts that they had expected. These consumers’ expectations had been formed from conversations that they had with consumers who had undertaken the SPS service before them. A few consumers also felt pressured to finalise their action plans within the specified time periods (e.g., before the end of the financial year) that had been established to assure the availability of funding. In these circumstances, the Guides worked with consumers to manage their disappointment and/or feelings of stress. Despite these initial challenges consumers seemed to encounter experiences that were beneficial to them, their families, and potentially their community, and these are discussed fully elsewhere [28].

The Guides’ reflections and consumers’ financial data showed that the expenditure of the individualised funds, despite some initial challenges, was managed well by all consumers. At the time of the study, although total allocated individualised funds of all consumers amounted to $76,859, total expenditure across consumers remained modest with approximately $52,000 being spent. This modest spending seemed related to some consumers delaying the implementation of their action plans due to unpredictable events (e.g., ill-health, delay in accessing their money, and/or residential challenges) or most consumers learning and/or practicing effective budgeting as highlighted in these statements:

“…opened separate bank accounts…money…easier to keep track of…” (C1)

“…proud of my budgeting…only bought what’s on…action plan…kept…receipts” (C8)

### Over-arching category 2 - Consistent contact in shared management and person-centred relationships enhanced the provision of timely and meaningful staff support to consumers

Overall, the support received by consumers, from working in shared management and person-centred relationships with the Guides, seemed essential to their recovery experiences that are fully discussed elsewhere [28]. The independent evaluator’s consumer interview (denoted IE C INT) and ten consumers’ action plans, questionnaires and speeches data indicated that the Guides’ and others’ support was highly valued. This support seemed to facilitate consumers’ self-direction of services and/or management of life demands, despite some consumers feeling uncertainty prior to beginning the SPS service, as highlighted in these statements:

“…support received helped participants to clarify what they wanted to achieve not only in the project but in their life as a whole.” (IE C INT)

“…participants found strength to continue on due to the support they received.” (IE C INT)

“It takes a bit of getting used to all this freedom of choice… feel like I am listened to…supported with what I want to achieve…Guide support has been great…helped me plan my goals…kept me on track…when it got hard.. helped me think of ways around things” (C1)

“…thought this was just going to be the same as other [services]…was very different…huge support network…facilitators, the Guides, and support worker. Without this support I would not have got through it....” (C9)

Over-arching category 2 was developed from 516 statements that revealed 1,891 descriptors, which were shared across four subcategories (second tier). The four subcategories (second tier) were: 1) who provided support to consumers; 2) the nature of support interactions; 3) the type of support provided; and 4) engagement of other support activities. The nature of support interactions was underpinned by three subcategories (third tier) including: 1) consistency in contact; 2) direct contact; and 3) attuned and responsive support. The type of support provided was established on two subcategories (third tier) including: 1) practical support; and 2) emotional support. The engagement of other support activities was established on two subcategories (third tier) including: 1) facilitating connections between consumers and others; and 2) managing aspects of the service.

The Guides’ reflections and/or meeting minutes, the independent evaluator’s consumer interview data and data relating to hours of support provided indicated that a range of people supported consumers. The Guides provided the strongest support to all consumers. The consumers also had access to support from family, friends, support workers, PCP facilitators, and health care professionals and groups in the community; however this could not be fully explored due to the limits of the data.

The nature of the support provided to consumers by the Guides involved having consistent contact with them and being available to them during and outside business hours. In this, the Guides provided regular direct contact for a number of reasons. They met (mostly) or spoke over the phone (occasionally) to discuss and/or self-direct (e.g., take actions) service or recovery activities (e.g., budgeting, SPS service related questionnaires, preparing job or course registration applications, preparing speeches, etc.…), celebrate successes, and/or work through challenges. All consumers received the most consistent and direct contact from the Guides during their weekly visits where they worked together to develop and/or begin the implementation of their action plans. On average, the Guides provided 5.5 hours of direct contact to each consumer every week for the duration of the SPS service; but this varied across consumers dependent on their needs. In times of high stress (e.g., loss of a loved one, going through surgery, or recovering from an accident), throughout the entirety of the SPS service, the Guides increased their contact with consumers as needed. The Guides also provided one consumer regular and strong levels of support for the duration of the SPS service. The contact between the Guides and a few consumers from the first group continued after they had completed the SPS service.

The data also showed that the Guides (denoted G) provided attuned and responsive support to consumers’ needs. In this, the Guides, working in shared management and person-centred relationships with consumers, maintained awareness of consumers’ unique needs, openly discussed challenges, celebrated achievements, and facilitated and empowered consumers to make their own choices and decisions along their recovery journey. Throughout the SPS service, the Guides’ attuned and responsive support seemed to enable consumers to address and work through various challenges and develop solutions that met their unique needs.

The data also showed that the types of support given by the Guides included both practical and/or emotional, as highlighted in these statements:

“Reunion planning…reflecting on Consumer 3’s journey…help…put together…story.” (G)

“Support…encouragement to Consumer 3 to [manage]…issues with oldest son” (G)

“Attended counselling session with Consumer 6…” (G)

“Spoke to Consumer 15 about…being honest…in regards to [their] wellbeing.” (G)

Practical support involved the Guides working with consumers to access to recovery goods and/or services. This ranged from researching and sourcing recovery resources (e.g., equipment), attending appointments and developing documentation (e.g., job applications or speeches). This also involved navigating through Internet sites (e.g., employment or community service sites), and learning strategies to manage interpersonal conflicts, personal challenges, and/or budgeting responsibilities. The Guides also provided consumers with general and specific emotional support. General emotional support involved validating experiences and/or talking through challenges. Specific emotional support provided to consumers involved encouraging them to take action, praising them for their achievements, and/or reflecting with them on the impact of particular successes and/or challenges. Most consumers’ speeches data showed that this support was essential to their recovery journey, as stated:

“…guide support has been great, because [Guide] helped me plan my goals and kept me on track…encouraged me when it got hard and helped me think of ways around things.” (C1)

“When I haven’t known how to handle a situation with the boys, I air it with my [Guide] …they not only validate how I feel but also offer me different tactics to approach the situation.” (C3)

The data also showed that as consumers progressed through the SPS service, most became self-reliant and less reliant on the Guides’ support. Three consumers needed less support than others once they started implementing their action plans. Most consumers, as they became familiar and/or more confident in pursuing their recovery goals (e.g., sourcing and procuring recovery resources, attending appointments and/or budgeting), became more self-sufficient. As consumers’ capacity to self-direct services grew, the Guides provided less support (e.g., home visits, attending with consumers organised meetings such as PCP reunion meetings, events/appointments), gradually moving from weekly to fortnightly and then monthly contact. In doing this, they responsively and consciously worked towards developing consumers’ autonomy, as highlighted in these statements:

“Started withdrawing…hands on support [to Consumer 3]… [as they are] managing quite well” (G)

“Encouraged Consumers 8 to problem solve… [generate] options… did not jump in and fix…” (G)

“Got Consumer 13 to do her own research on prices” (G)

In reducing support to consumers, the Guides engaged in other support activities. This involved them communicating regularly with support workers, and others within (e.g., family) and outside the services (e.g., community professionals and/or organisations). This was done to enhance others’ awareness of consumers’ needs and facilitate connections between them and the consumers. The Guides also managed service aspects to support both the consumers’ and the service provider’s needs (e.g., facilitating the timely allocation of funds, reconciling expenditure, building strong inter-service network connections and relationships for consumers potential use). The other support activities, provided by the Guides, optimised consumers’ chances to achieve their recovery goals, as highlighted in these statements:

“Spoke to support worker …helped…identify… training needs.” (G)

“Got support worker to focus…on [developing] literacy skills with Consumer 1.” (G)

“…working with coordinator and family to have Consumer 4 reinstated into…” (G)

“Worthwhile…to ‘bulk buy’…better discounts…stretch…money further.”(G)

### Over-arching category 3 - High quality shared management and person-centred relationships with staff and the opportunity to self-direct services enabled consumers’ change and growth

Over-arching category 3 is highly related to, but independent of, the previous over-arching category in that it emerged from the process of interaction, which occurred via the consistent and direct contact that took place between consumers and the Guides. The Guides’ reflections and the independent evaluator’s consumer interview and report data revealed a total of 1,304 descriptors, developed from 684 statements. This over-arching category was formed from two subcategories (second tier) that included the 1) consumers’ characteristics; and 2) the Guides’ characteristics. These two subcategories were developed from subcategories (third tier) that were initially coded as a range of person related characteristics (Table 5). The data suggested that the interaction of person characteristics between the consumers and Guides, while they engaged in shared management and person-centred relationships, seemed to enable high quality relationship encounters and enhance consumers’ self-direction and recovery experiences that are fully discussed elsewhere [28].

**Table 5:** Consumers’ and the guides’ person characteristics that enabled high quality relationships

**Consumers**			**Guides**		
**Characteristics identified in the data**	**Number of contributions**		**Characteristics identified in the data**	**Number of contributions**	
	**Consumers**	Descriptors*		**Consumers**	Descriptors*
Courage and commitment	16	160	Awareness, knowledge, and ability to educate	5	181
Awareness, flexibility, and initiative	16	118	Support and contribution	8	170
Honesty and openness	13	105	Initiative and commitment	3	156
Sense of humour	6	13	Honesty, courage, and openness	3	150
Loyalty and compassion	3	9	Encouragement, guidance, and/or the ability to develop effective strategies	6	121
			Sensitivity	6	71
**Total descriptors**		455*	**Total descriptors**		849*

*Descriptors developed using the consumers’, Guides’ and independent evaluator’s data.

Consumers’ characteristics were developed using the independent evaluator’s interview and report, the Guides’ reflections, and the consumers’ questionnaires and speeches data. The Guides’ characteristics were developed using the independent evaluators’ interview, the Guides’ reflections, and some consumers’ MIC questionnaires and speeches data.

All consumers’ showed some or many of the characteristics outlined in Table 5. These seemed present in their behaviour and ways of thinking while they were self-directing their services and recovery. These characteristics seemed to support them to achieve their recovery goals and develop their capacity to self-direct, as highlighted in these statements:

“…get myself into bad situations when I get…angry…really want to change…” (C1)

“…agreed to do the [SPS service]…was excited…anxious…uncertain…found out more about me…my world is opening up… (C3)

“…was a bit hesitant…thought…just…the same as other programs…realized…was very different…helped me…think about a positive new life for me and how to get there. (C9)

“…was scary… made me feel like I wasn’t alone…learnt…to make conversation…that it is okay to set goals and dreams.” (C10)

“…found it very strange…hard…finally clicked…made sense…could see others seeing me in a different light…slowly started to believe in myself…see myself as worthy......have a purpose, a journey to partake.” (C14)

More specifically, consumers’ openness to look at themselves and their flexibility and courage to attempt and engage (even when lacking confidence or certainty) new learning when having to self-direct services and resolve challenges supported them. They learned and/or experienced themselves and/or others in new or different ways. Their willingness to engage in new learning (e.g., ways of thinking and acting) provided them insight into their strengths, successes and limitations. These experiences alongside the support from the Guides enabled them to stay motivated to persevere (despite some initial negative feelings and/or struggling at times). The Guides’ characteristics (Table 5) seemed to enable them to become and/or stay mindful of consumers’ needs, and empowered them to provide effective shared management and person-centred relationships and timely, attuned, and responsive and meaningful support, as highlighted in these statements:

“Spoke to Consumer 1 [about]…loss of motivation at the moment due to ill health.” (G)

“Supported Consumer 2 through family crisis.” (G)

“Praised Consumer 6 [for their] efforts to address this” (G)

“…honest conversations, creating trust…reiterating Consumer 7 will not let us down.” (G)

“…not a one size fits all…some…needed extra support…other[s]…self-drive…minimal support” (G)

More specifically, the Guides’ provision of support to assist consumers to self-direct seemed enabled by their personal characteristics. The Guides’ initiative and commitment enabled them to provide timely encouragement and guidance that supported consumers. Their awareness and ability to educate consumers on strategies that might be helpful to them, and communicate with honesty, courage, and openness enabled them to provide meaningful support. This was balanced with the showing of sensitivity, respect and understanding towards consumers. This allowed for consumers’ lived experiences (e.g., challenges with services and/or life) to be discussed, and for them to experience effective support to achieve self-direction and progress their recovery journey at a pace and in a manner that met their unique needs.

In the shared management and person-centred relationships, the consumers’ and Guides’ person characteristics seemed to interact in an attuned manner and complement each other. This attuned interaction appeared to create a safe and trusted working space that enabled for high quality interpersonal relationship experiences. In these relationships, consumers encountered new or irregular experiences, including feeling respected, valued, understood, and trusted. This promoted, at varied levels, their feelings of happiness, confidence, worthiness, and self-pride and enhanced their self-direction and recovery experiences, fully discussed elsewhere [28], as highlighted in these statements:

“…Guide…great…helped me plan…kept me on track…” (C1)

“… [staff]…guided…showed..[.us] we are valued…discovered things about me that I couldn’t see…” (C2)

“… [outcomes] come....from…respect, understanding…support… received from [staff]…” (C3)

“…proved…people…care about my future…willing to help…never happened…in my life…” (C8)

“…staff helped…encouraged…believed in me…all the way…provided… the tools… to get out of that hole…filled me with confidence…can’t thank them enough.” (C9)

These three over-arching categories (first tier), and related subcategories (second and third tiers) have been reported separately for the purpose of the study; but constant comparisons during coding and analysis revealed that these categories and subcategories were inter-related. These results showed that access to individualised funds and the chance to experience high quality shared-management and person-centred relationships and self-direction were essential for the progress of consumers’ self-direction and recovery. The individualised funds enabled consumers’ timely access to recovery resources. The experience of shared management and person-centred relationships, while self-directing their services, provided them with essential practical and emotional support. In this, consumers experienced positive and/or corrective emotional encounters that facilitated their learning, insight, change and growth, enabling them to gain hope for a happier future. Thus, the impact of the SPS service on the lived experiences of consumers seemed related to the combined effects of having access to the SPS service components and the quality of experience of these components.

## Discussion

These consumers’ lived experiences showed that access to individualised funds provided them benefit; although some consumers initially struggled to effectively manage their budgets. This result is congruent with the suggestion made by others that consumers may encounter greater demand and responsibility to manage funds and service aspects [13,21]. In this study all but one of the consumers, who had struggled, overcame such challenges with the support of the Guides provided through consistent and high quality shared management and person-centred relationships. These results are congruent with the results of other research [1,13,15,21,27] that showed consumers spent funds well, and modestly, to procure recovery resources to meet their needs across all aspects of their lives. Most of these consumers had limited access to employment and income. Having access to individualised funds to procure necessary resources provided them with chances that may have taken longer or may not have been possible to experience without access to individualised funds, as has been suggested in other research [1]. As noted by others [1,13,15,20,21,27], access to individualised funds enabled consumers’ timely access to recovery resources. This timely access provided them with positive emotional experiences that seemed to motivate and mobilise them, facilitating change and influencing their recovery experiences [28]. Thus, the SPS service seemed to enable consumers to self-direct their recovery journey across all aspects of their life, and not just in terms of their mental illness. This improved their lived experiences, enabling them to start building a life and future that was meaningful and hopeful, supporting them to live as best as they could with and beyond their mental illness [12,28].

As with previous findings and suggestions, these consumers’ lived experiences showed that they benefitted from encountering consistent and high quality shared management and person-centred relationships with the Guides throughout, and for some beyond the SPS service period [1,10,13,15,20,27]. This study’s results support the suggestions made by others in that the Guides’ consistent contact with consumers seemed to enable the Guides to gain a clear and/or a more holistic understanding of consumers’ lives and needs [10]. From this understanding, the Guides seemed empowered to provide attuned, responsive and meaningful practical and emotional support. This enabled the support to be tailored to, and specifically meet, consumers’ unique needs. As noted in other research, such support was essential for these consumers to effectively develop and implement their action plans [15]. Although there were individual differences in the level of support needed by consumers, these results showed that tailoring support to specifically meet each consumer’s unique needs and challenges enabled them to progress. Most consumers’ capacity to self-direct their services, at varied levels, was enhanced. The results of this study showed that in SPS services, as also noted by others, the interaction of person related characteristics impacted on the quality of the relationship formed [23-25,27]. Unlike other SPS research cited in this study, these results closely explored the person characteristics of consumers and the Guides. In this, the complimentary nature of the interaction of these characteristics that emerged seemed essential for consumers’ growth of their self-directing capacity, recovery, and life experiences [28].

Overall, these results suggest that access to SPS services provided consumers with the opportunity to experience life in ways that was different to what they had been previously experienced. These consumers had not had the chance to consider living their lives beyond aspects of their mental illness. Moreover, they had not had the chance to access desired recovery resources in a timely manner, or experience high quality support relationships. Further, none of them had experienced freedom to actively drive, decide and make choices across all aspects of their recovery, as well as plan their life dreams. From this experience, most consumers had started to develop new ways of thinking and behaviour, having encountered new and positive emotional experiences. This seemed to facilitate their change and growth and achievement of positive recovery experiences [28]. Within these experiences, their views of themselves and connections with others improved, which enhanced their experiences with the service as a whole, staff, family and/or friends, and individuals and organisations in the community [28]. Such experiences may not have been encountered had these services being underpinned by a biomedical model [6,7,10].

Together, these results suggested that the successful undertaking of SPS services by consumers is reliant on the presence of three combined factors including having: 1) access to individualised funds; 2) consistent contact in shared management and person-centred relationships; and 3) high quality shared management and person-centred relationships and the chance to self-direct. Within this, it is tentatively suggested that success of these consumers undertaking of SPS services was reliant on the goodness of fit between the person related characteristics of consumers and the Guides, which seems to be founded on the strength of the Guides’ characteristics. Other research has noted the value consumer-staff relationships within SPS services [1,13,15,27]; but none was found to focus closely on the characteristics underpinning these interpersonal relationships and their impact on consumers’ lived experiences.

Despite these results, it is essential to recognise that the impact of SPS services needs further exploration and validation to overcome the limitations of this study, which was based on data collected in the past from a self-selected and small sample. Reliance on data collected in the past limited the exploration of experiences outside that data (e.g., the challenges experiences by some consumers). It is essential to gain a more balanced and clearer understanding of the impact of SPS services using a broader scope of data. For example, collecting data in real-time from consumers around a range of related experiences would enable this. Data may be collected on consumers’ challenges with the SPS service or other types of personal (e.g., from support workers, family and friends), and /or community (e.g., counselling) support that they may be engaging alongside the SPS service. Such information would assist to develop a more holistic understanding of the impact of SPS services on the lived experiences of consumers. Thus, the transferability of these results is limited.

The self-selected and small sample size also limited the transferability of these results. This sample may have possessed unique qualities that may have influenced the results, alongside or independent of the impact of the SPS service. For example, the positive experiences and achievements that most consumers achieved may a consequence of their positive pre-service experiences with the service provider, rather than the SPS service. Further, as with any qualitative analyses, the data coding was reliant on the interpretation of coder. Although potential coding subjectivity was minimised by using rigorous methodology (e.g., using a systematic, consistent and all-encompassing coding approach, and the thorough quality checking of coding throughout) the use of more expanded research methods would strengthen insight into the impact SPS services on consumers’ lived experiences. This would enable for associated factors (e.g., consumers’ and the support person’s relational patterns and experiences and characteristics and abilities), corrective emotional experiences and change to be understood with more clarity, certainty and depth.

## Conclusions

The use of individualised funds, shared management and person-centred relationships, and the chance to self-direct services enhanced most consumers’ lived experiences. These SPS service enabled staff to provide, and consumers to experience, timely access to recovery resources, consistent contact, and attuned and responsive high quality support. In this, consumers experienced meaningful services that supported their change and growth. The impact of the SPS service seemed founded on the goodness of fit between person characteristics of the Guides and consumers that enabled consumers to experience corrective emotional experiences, move towards achieving desired recovery experiences and building a meaningful and hopeful life to live with and beyond their mental illness.

## Competing interest

The authors declare that they have no competing interests.

## Authors’ contributions

AB conceived and designed the study, and AB and SP coordinated and implemented the study. SP conducted the day-to-day management of data collection and collation, and coded the data for analysis. SP and AB analysed the data and developed the preliminary and final pilot model of service practice. All authors contributed to the preparation and revision of the draft of this manuscript, and have approved the final manuscript for publication.

## Authors’ information

(1) Sunila Peterson Provisionally Registered Psychologist and Research Associate in the School of Occupational Therapy and Social Work (2) Associate Professor Angus Buchanan Occupational Therapist and Deputy Head of the School of Occupational Therapy and Social Work, Curtin University (3) Professor Torbjorn Falkmer Occupational Therapist and Senior Research Fellow in the School of Occupational Therapy and Social Work.

## Supplementary Material

Additional file 1 Description of data documents provided to researchers by three data sources. Click here for file

Additional file 2 Number and percentage of data documents received and analysed. Click here for file
