# Supplementary material for: The impact of services that offer individualised funds, shared management, person-centred relationships, and self-direction on the lived experiences of consumers with mental illness
Source: Int J Ment Health Syst. 2014 Jun 3;8:20. doi: 10.1186/1752-4458-8-20 (PMC4061914; doi:10.1186/1752-4458-8-20)
Supplement: Additional file 1 — Description of data documents provided to researchers by three data sources. [file 1752-4458-8-20-S1.doc]

**Additional file 1: Description of data documents provided to researchers by three data sources**

| Data sources | Documents (developed in the past) | Description of information |
| --- | --- | --- |
| Independent evaluator  Evaluator assessing the PCP* component of the SPS service (data compiled in the past on consumers’ past and current experiences) | Consumer interview (past** and current*** experiences) reports | Consumers’ interview data (e.g., questions, responses, and summarising) of reflections since undertaking the SPS service were collated into three full and summary reports |
|  | PCP evaluation reports | Evaluation reports provided to the organisation reflecting the evaluators’ interpretation of consumer s’ and staffs’ data in relation the to their undertaking of the PCP component of the SPS service |
|  | Staff interviews (past and current experiences) | Staffs’ interview data collated into reports based on the Guides’ and other staffs’ reflections |
| The Guides  Staff who supported consumers’ throug**h** shared management and person-centred relationships across all phases of the SPS service. Data (documented in the past) related to the consumers’ progress and program implementation experiences in the past (retrospective) and at the time (current) | Personal learning (past experiences) | Guides’ reflections on their learning of service administration and process (e.g., how best to support consumers to enable their achievement of recovery goals and self-direction) |
|  | Organisational reports (past and current experiences) | Reports provided to key organisational stakeholders, at irregular meetings, on the progress of the consumers |
|  | Reflections on consumers  (past and current experiences) | Guides’ reflections on what was done, what worked, what could improve, and the next steps in relation to consumers’ achievement of recovery goals and self-direction completed up to 4-7 times |
|  | Reflections on the services  (past and current experiences) | Guides’ reflections on what was done, what worked, what could improve, and the next steps in relation to their administration of services to support consumers through the SPS service |
|  | Speeches  (past and current experiences) | Speeches prepared and presented by the Guides for reunion events that capture the Guides’ experiences working with the participants and the SPS service |
| Consumers  Consumers who undertook the SPS service in three groups of 5 – 6 members about six months apart, over a total period of 16 months. Data (documented in the past) related to consumers’ experiences in the past (retrospective) and at the time (current) | Action plans (current experiences) | Fine-tuned from consumers’ dreams identified during PCP process. These defined their recovery goals including: change they wanted to achieve; the first steps they need to take; who they want to work with; when they want to take the first steps; and the costs that will be involved. Recovery goals on any action plan ranged from 4 - 10 |
|  | Most important changes (MIC) in my life questionnaire (current experiences) | Open and closed ended questioned forms that were completed for the independent evaluator by consumers 1-4 times per group whilst undertaking the SPS service that described at that time: their mental health and life experiences; and supports or barriers to their recovery |
|  | Recovery questionnaire  (past and current experiences) | A short open and closed ended questioned form completed for the organisation by consumers that described: their history; people and factors that may help them on their recovery journey; who they do not want involved in their recovery; signs when they know they are becoming unwell; and actions they can take to help themselves if they became or prevent them from becoming unwell |
|  | Speeches (past and current experiences) | A presentation developed by consumers describing their life experiences including challenges, changes, outcomes and benefits since undertaking the SPS service |

*PCP – person centred planning| **past experience– retrospective data |*** current experience – occurring at the time of data collection
