# Supplementary material for: The impact of services that offer individualised funds, shared management, person-centred relationships, and self-direction on the lived experiences of consumers with mental illness
Source: Int J Ment Health Syst. 2014 Jun 3;8:20. doi: 10.1186/1752-4458-8-20 (PMC4061914; doi:10.1186/1752-4458-8-20)
Supplement: Additional file 2 — Number and percentage of data documents received and analysed. [file 1752-4458-8-20-S2.doc]

**Additional file 2: Number and percentage of data documents received and analysed**

| **Data sources and types of de-identified data** | | **Total # received** | | **Percentage coded** | |
| --- | --- | --- | --- | --- | --- |
| **Data sources** | **Data types** | **Documents** | **Pages** | **Documents** | **Pages** |
| Independent evaluator’s data  (documented in the past for PCP evaluation | Consumer interviews (summaries) | 3 | 3 | 100.00% | 100.00% |
|  | Consumer interviews (full reports) | 3 | 35 | 66.66% | 62.85% |
|  | Reports | 3 | 37 | 100.00% | 100.00% |
|  | Staff interviews | 2 | 16 | 100.00% | 100.00% |
|  | **Subtotal** | **11** | **91** | **90.91%** | **85.71%** |
|  |  |  |  |  |  |
| The Guides’ data  (documented in the past) | Personal learning | 2 | 2 | 100.00% | 100.00% |
|  | Organisational reports | 7 | 7 | 100.00% | 100.00% |
|  | Reflections on consumers progress  Across four questions   1. What did I do* 2. What worked well* 3. What could I do differently* 4. Where to next*   Across the three groups*  (4 - 7 times per group) | 292 | 292* | 83.90% | 83.90% |
|  | Reflections on four service phases  Across four areas   1. Selection phase 2. Administration of PCP** 3. Development of AP*** 4. Implementation of AP     Four questions asked under each area   1. What did I do* 2. What worked well* 3. What could I do* differently 4. Where to next* | 53 | 53* | 28.30% | 28.30% |
|  | PCP reunion speeches | 4 | 9 | 100.00% | 100.00% |
|  | **Subtotal** | **358** | **363** | **76.26%** | **76.58%** |
| Consumers’ data  (documented in the past) | Action plans | 16 | 23 | 100.00% | 100.00% |
|  | Most important change (MIC) in my life questionnaire (1-4 times per group) | 51 | 102 | 100.00% | 100.00% |
|  | Recovery questionnaire | 16 | 160 | 100.00% | 100.00% |
|  | PCP reunion speeches | 21 | 21 | 100.00% | 100.00% |
|  | **Subtotal** | **104** | **306** | **100.00%** | **100.00%** |
|  | **Grand total** | **473** | **760** | **81.81%** | **87.10%** |

*Data only a quarter of a page | **Person centred planning (PCP) | *** Action plan (AP)
